# Supplementary material for: Nicotinamide Mononucleotide Supplementation Improves Mitochondrial Dysfunction and Rescues Cellular Senescence by NAD+/Sirt3 Pathway in Mesenchymal Stem Cells
Source: Int J Mol Sci. 2022 Nov 25;23(23):14739. doi: 10.3390/ijms232314739 (PMC9738479; doi:10.3390/ijms232314739)
Supplement: Supplementary file 1 [file ijms-23-14739-s001.zip › ijms-1974305-supplementary.pdf]

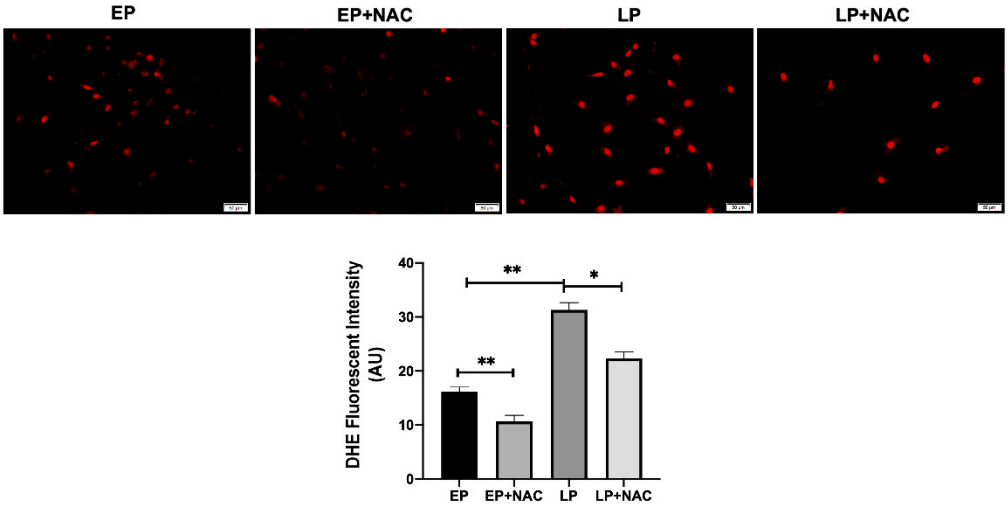

Figure S1. Treatment with N-Acetyl-cysteine (NAC) decreases reactive oxygen species (ROS) levels in early and late passage mesenchymal stem cells (MSCs). The ROS levels determined by dihydroethidium (DHE) staining (scale bar =50μm). Data are expressed as mean ± SD, n=3, *P<0.05, **P<0.01.
